# Supplementary material for: Excitatory cholecystokinin neurons in the CA3 area regulate the navigation learning and neuroplasticity
Source: eLife. 2026 Jul 28;14:RP109001. doi: 10.7554/eLife.109001 (PMC13412322; doi:10.7554/eLife.109001)
Supplement: Peer review history

# Editor report: eLife Assessment

**DOI:** 10.7554/eLife.109001.3.sa0

**Authors:** Helen E Scharfman

**Affiliations:** Nathan Kline Institute United States

**Keywords:** Incomplete, Valuable

This study presents data suggesting that excitatory cholecystokinin (CCK)-expressing neurons in hippocampal area CA3 influence hippocampal-dependent memory using multiple methods to manipulate excitatory CCK-expressing CA3 neurons. The study is **valuable**, particularly considering that most past studies of CCK-expressing neurons have focused on those neurons that co-express CCK and GABA. Currently, the strength of evidence is **incomplete**, but it would improve if evidence of specificity were provided and other concerns were addressed. If this is not possible, the conclusions, particularly those requiring evidence of specific targeting of excitatory neurons, should be modified accordingly.

# Referee report: Reviewer #1 (Public review)

**DOI:** 10.7554/eLife.109001.3.sa1

Summary:

CCK is the most abundant neuropeptide in the brain, and many studies have investigated the role of CCK and inhibitory CCK interneurons in modulating neural circuits, especially in the hippocampus. The manuscript presents interesting questions regarding the role of excitatory CCK+ neurons in the hippocampus, which has been much less studied compared to the well-known roles of inhibitory CCK neurons in regulating network function. The authors adopt several methods including transgenic mice and viruses, optogenetics, chemogenetics, RNAi, and behavioral tasks to explore these less-studied roles of excitatory CCK neurons in CA3. They find that the excitatory CCK neurons are involved in hippocampal-dependent tasks such as spatial learning and memory formation, and that CCK-knockdown impairs these tasks.

However, these questions are very dependent on ensuring that the study is properly targeting excitatory CCK neurons (and thus their specific contributions to behavior).

There needs to be much more characterization of the CCK transgenic mice and viruses to confirm the targeting. Without this, it is unclear whether the study is looking at excitatory CCK neurons or a more general heterogeneous CCK neuron population.

Strengths:

This field has focused mainly on inhibitory CCK+ interneurons and their role in network function and activity, and thus this manuscript raises interesting questions regarding the role of excitatory CCK+ neurons, which have been much less studied.

Weaknesses:

(1a) This manuscript is dependent on ensuring that the study is indeed investigating the role of excitatory CCK-expressing neurons themselves and their specific contribution to behavior. There needs to be much more characterization of the CCK-expressing mice (crossed with Ai14 or transduced with various viruses) to confirm the excitatory-cell targeting. Without this, it is unclear whether the study is looking at excitatory CCK neurons or a more general heterogeneous CCK neuron population.

(2) The methods and figure legends are still extremely sparse, still leading to many questions regarding methodology and accuracy. More details would be useful in evaluating the tools and data, and the lack of proper quantification is still prevalent throughout the paper. In many places, only % values are noted, or only images are presented, and the number of cells counted is almost never reported.

# Referee report: Reviewer #2 (Public review)

**DOI:** 10.7554/eLife.109001.3.sa2

Summary:

In this study, the authors have demonstrated, through a comprehensive approach combining electrophysiology, chemogenetics, fiber photometry, RNA interference, and multiple behavioral tasks, the necessity of projections from CCK+ CAMKIIergic neurons in the hippocampal CA3 region to the CA1 region for regulating spatial memory in mice. Specifically, authors have shown that CA3-CCK CAMKIIergic neurons are selectively activated by novel locations during a spatial memory task. Furthermore, authors have identified the CA3-CA1 pathway as crucial for this spatial working memory function, thereby suggesting a pivotal role for CA3 excitatory CCK neurons in influencing CA1 LTP. The data presented appear to be well-organized and comprehensive.

Strengths:

(1) This work combined various methods to validate the excitatory CCK neurons in the CA3 area; these data are convincing and solid.

(2) This study demonstrated that the CA3-CCK CAMKIIergic neurons are involved in the spatial memory tasks; these are interesting findings, which suggest that these neurons are important targets for manipulating the memory-related diseases.

(3) This manuscript also measured the endogenous CCK from the CA3-CCK CAMKIIergic neurons; this means that CCK can be released under certain conditions.

Weaknesses:

In summary, this work can be formally accepted after the revision. For the limitations of the revision, the distinct neural effects of cholecystokinin (CCK) receptors (CCK-1R, CCK-2R, and CCK-3R) on hippocampal function have not been fully elucidated. Recent studies indicate that CCK-2R can modulate hippocampal activity at CA3-Schaffer collateral synapses; however, the roles of CCK-1R and CCK-3R in hippocampal function remain poorly characterized, with limited experimental evidence supporting their involvement. Overall, this study provides an interesting and novel perspective on the role of excitatory CCK signaling in hippocampus-dependent navigation learning.

# Referee report: Reviewer #3 (Public review)

**DOI:** 10.7554/eLife.109001.3.sa3

Summary:

Fengwen Huang et al. used multiple neuroscience techniques (transgenetic mouse, immunochemistry, bulk calcium recording, neural sensor, hippocampal-dependent task, optogenetics, chemogenetics, and interfer RNA technique) to elucidate the role of the excitatory cholecystokinin-positive pyramidal neurons in the hippocampus in regulating the hippocampal functions, including navigation and neuroplasticity.

Strengths:

(i) The authors provided the distribution profiles of excitatory cholecystokinin in the dorsal hippocampus via the transgenetic mice (Ai14::CCK Cre mice), immunochemistry, and retrograde AAV.

(ii) The authors used the neural sensor and light stimulation to monitor the CCK release from the CA3 area, indicating that CCK can be secreted by activation of the excitatory CCK neurons.

(iii) The authors showed that the activity of the excitatory CCK neurons in CA3 is necessary for navigation learning

(iv) The authors demonstrated that inhibition of the excitatory CCK neurons and knockdown of the CCK gene expression in CA3 impaired the navigation learning and the neuroplasticity of CA3-CA1 projections.

Weaknesses:

(i) The causal relationship between navigation learning and CCK secretion remains nebulous; answering this question will require a more sensitive CCK-BR sensor in future work.

# Author comment: Author response

**DOI:** 10.7554/eLife.109001.3.sa4

**Authors:** Fengwen Huang, Abdul Baset, Stephen Temitayo Bello

**Affiliations:** City University of Hong Kong Hong Kong Hong Kong; City University of Hong Kong Hong Kong Hong Kong; City University of Hong Kong Hong Kong United States

The following is the authors’ response to the original reviews.

**Public Reviews:**

**Reviewer #1 (Public review):**

Summary:

CCK is the most abundant neuropeptide in the brain, and many studies have investigated the role of CCK and inhibitory CCK interneurons in modulating neural circuits, especially in the hippocampus. The manuscript presents interesting questions regarding the role of excitatory CCK+ neurons in the hippocampus, which has been much less studied compared to the well-known roles of inhibitory CCK neurons in regulating network function. The authors adopt several methods, including transgenic mice and viruses, optogenetics, chemogenetics, RNAi, and behavioral tasks to explore these less-studied roles of excitatory CCK neurons in CA3. They find that the excitatory CCK neurons are involved in hippocampal-dependent tasks such as spatial learning and memory formation, and that CCK-knockdown impairs these tasks.

However, these questions are very dependent on ensuring that the study is properly targeting excitatory CCK neurons (and thus their specific contributions to behavior). There needs to be much more characterization of the CCK transgenic mice and viruses to confirm the targeting. Without this, it is unclear whether the study is looking at excitatory CCK neurons or a more general heterogeneous CCK neuron population.

Strengths:

This field has focused mainly on inhibitory CCK+ interneurons and their role in network function and activity, and thus, this manuscript raises interesting questions regarding the role of excitatory CCK+ neurons, which have been much less studied.

Weaknesses:

(1a) This manuscript is dependent on ensuring that the study is indeed investigating the role of excitatory CCK-expressing neurons themselves and their specific contribution to behavior. There needs to be much more characterization of the CCK-expressing mice (crossed with Ai14 or transduced with various viruses) to confirm the excitatory-cell targeting. Without this, it is unclear whether the study is looking at excitatory CCK neurons or a more general heterogeneous CCK neuron population.

Thank you for this constructive comment. Indeed, the current study lacks comprehensive strategies to unequivocally distinguish excitatory CCK neurons from heterogeneous CCK neuronal populations. Nevertheless, we provide multiple lines of evidence supporting the distribution of CaMKIIα/Vglut1-expressing CCK^+^ neurons in the hippocampus (Figure 1F), using complementary approaches including transgenic mouse models as well as viral and antibody-based labeling (Figure 1A, Figure 1H-I). In addition, we demonstrate that 635 nm light reliably evokes field excitatory postsynaptic potentials (fEPSPs) at CA3-Schaffer collateral synapses expressing DIO-CaMKIIα-ChrimsonR in vitro (Figure 2A-F). Importantly, these light-evoked excitatory synaptic responses are abolished by AMPA and NMDA receptor antagonists (CNQX and APV), confirming the excitatory nature of the DIO-CaMKIIα-ChrimsonR-expressing synapses. To demonstrate the future works that can further support our findings and conclusions, we have added the strategies that can be conducted in the Discussion section in the revision:

“Due to technical limitations at the current stage, we were unable to perform whole-cell recordings or pharmacological manipulations using CCK receptor antagonists. In future studies, the application of these approaches to directly record and selectively block EPSPs from excitatory CCK neurons in the hippocampus will further strengthen and validate our conclusions.” (Line 265 - line 269 in the revision).

(1b) For the experiments that use a virus with the CCK-IRES-Cre mouse, there is no information or characterization on how well the virus targets excitatory CCK-expressing neurons. (Additionally, it has been reported that with CaMKIIa-driven protein expression, using viruses, can be seen in both pyramidal and inhibitory cells).

We thank the reviewer for this insightful comment regarding the specificity of viral targeting in CCK-IRES-Cre mice.

To address this concern, we performed additional characterization of viral expression in CA3. We found that DIO-CaMKIIα-mCherry expression showed a high degree of colocalization with CaMKIIα immunoreactivity, indicating preferential targeting of excitatory neurons (sFigure 1A-B; sFigure 2A-B; sFigure 3A-B). We showed an example to confirmed the high specificity of the AAV for infecting the excitatory CCK neurons in CA3 area.

Besides, we acknowledge prior reports showing that CaMKIIα-driven viral expression can, in some cases, be detected in a small subset of inhibitory neurons. However, because CA3-Schaffer collateral projections to CA1 arise exclusively from excitatory CA3 pyramidal neurons, any potential expression in inhibitory CCK^+^ interneurons are unlikely to directly contribute to the recorded CA1 synaptic responses in our electrophysiological experiments. That said, we cannot fully exclude the possibility that a minor population of inhibitory CCK⁺ neurons could indirectly modulate CA3 pyramidal neuron activity via local circuit mechanisms, particularly in experiments involving optogenetic manipulation or shRNA expression. We now explicitly acknowledge this limitation in the revised manuscript:

“Importantly, to further improve cell-type specificity, we propose an intersectional genetic strategy using CCK-IRES-Cre × VGlut1-Flp mice combined with a Cre-On/Flp-On (Con/Fon) AAV, which would restrict expression exclusively to excitatory CCK-expressing neurons and eliminate potential contributions from inhibitory CCK^+^ cells. This approach will be implemented in future studies to refine circuit specificity.” (Line 269 - line 273 in the revision).

(2) The methods and figure legends are extremely sparse, leading to many questions regarding methodology and accuracy. More details would be useful in evaluating the tools and data. More details would be useful in evaluating the tools and data. Additionally, further quantification would be useful-e.g. in some places, only % values are noted, or only images are presented.

Thank you for these constructive comments. We have expanded the methodological descriptions in both the Methods section and the figure legends to provide sufficient detail for evaluating the experimental tools and data accuracy. In addition, we have added quantitative analyses where previously only representative images or percentage values were shown. Specifically, quantification has now been included for each AAV condition in the corresponding figures in the revised manuscript.

(3) It is unclear whether the reduced CCK expression is correlated, or directly causing the impairments in hippocampal function. Does the CCK-shRNA have any additional detrimental effects besides affecting CCK-expression (e.g., is the CCK-shRNA also affecting some other essential (but not CCK-related) aspect of the neuron itself?)? Is there any histology comparison between the shRNA and the scrambled shRNA?

Recent studies from our lab demonstrated that knockout the CCK gene expression significantly attenuates the hippocampal-dependent spatial learning and CA3-CA1 LTP, indicating CCK plays a critical role in modulating the hippocampal functions[1,2]. Additionally, CCK-shRNA or CCK-scramble did not significantly affect the excitatory synaptic transmission in the CA3-CA1 projections, hinting that CCK-shRNA may exhibits no obvious adverse effect on other neural components.

Finally, we have provided the histology comparison between the shRNA and the scrambled shRNA regrading the expression level of the CCK protein (Pro-CCK) in the revision. Our result shows that CCK-shRNA (left panel) significantly reduced CCK expression in CA3^CCK^-positive neurons compared with the CCK-Scramble group (right panel).

Citation:

(1) Wang, J. L., Sha, X. Y., Shao, Y., Zhang, Z. H., Huang, S. M., Lin, H., ... & Sun, J. P. (2025). Elucidating pathway-selective biased CCKBR agonism for Alzheimer’s disease treatment. Cell.

(2) Zhang, N., Sui, Y., Jendrichovsky, P., Feng, H., Shi, H., Zhang, X., ... & He, J. (2024). Cholecystokinin B receptor agonists alleviates anterograde amnesia in cholecystokinin-deficient and aged Alzheimer's disease mice. Alzheimer's research & therapy, 16(1), 109.

https://doi.org/10.7554/eLife.109001.1.sa2

**Reviewer #2 (Public review):**

Summary:

In this study, the authors have demonstrated, through a comprehensive approach combining electrophysiology, chemogenetics, fiber photometry, RNA interference, and multiple behavioral tasks, the necessity of projections from CCK+ CAMKIIergic neurons in the hippocampal CA3 region to the CA1 region for regulating spatial memory in mice. Specifically, authors have shown that CA3-CCK CAMKIIergic neurons are selectively activated by novel locations during a spatial memory task. Furthermore, authors have identified the CA3-CA1 pathway as crucial for this spatial working memory function, thereby suggesting a pivotal role for CA3 excitatory CCK neurons in influencing CA1 LTP. The data presented appear to be well-organized and comprehensive.

Strengths:

(1) This work combined various methods to validate the excitatory CCK neurons in the CA3 area; these data are convincing and solid.

(2) This study demonstrated that the CA3-CCK CAMKIIergic neurons are involved in the spatial memory tasks; these are interesting findings, which suggest that these neurons are important targets for manipulating the memory-related diseases.

(3) This manuscript also measured the endogenous CCK from the CA3-CCK CAMKIIergic neurons; this means that CCK can be released under certain conditions.

Weaknesses:

(1) The authors do not mention which receptors of the CCK modulate these processes.

We appreciate the reviewer for raising this important question. Based on our recent work, CCK-B receptors are the primary neural components mediating CCK functions in the hippocampus at both the synaptic plasticity and behavioral levels (Su et al., 2023; Zhang et al., 2024; Wang et al., 2025). To clarify this mechanism, we have added the following content to the revised manuscript:

“Based on our recent work, CCK signaling in the hippocampus is predominantly mediated by CCK-B receptors, which play a critical role in regulating synaptic plasticity and spatial memory-related behaviors.” (Line 105 - line 106 in the revision).

(2) This author does not test the CCK gene knockout mice or the CCK receptor knockout mice in these neural processes.

Thank you for this insightful comment. We previously tested these experiments in an earlier study. Our results showed that high-frequency electrical stimulation failed to induce significant LTP in the CA3-CA1 pathway in both CCK gene knockout (CCK-KO) mice and CCK-B receptor knockout (CCK-BR-KO) mice in vitro (Su et al., 2023; Zhang et al., 2024; Wang et al., 2025). These findings indicate that CCK mediates its synaptic effects predominantly through CCK-B receptors in the CA3-CA1 pathway. Accordingly, we have added this description to the revised manuscript.

“Additionally, high-frequency electrical stimulation fails to induce LTP in the CA3-CA1 pathway in both CCK-KO and CCK-BR-KO mice, indicating that CCK-dependent synaptic plasticity in this circuit is primarily mediated by CCK-B receptors.” (Line 170 - line 173 in the revision).

(3) The author does not test the source of CCK release during the behavioral tasks.

We thank the reviewer for raising this important point. In our previous work, we directly monitored CCK release in the hippocampus during an object-exploration task using a GPCR-based CCK-BR sensor combined with fiber photometry (Su et al., 2023). During object exploration, we observed a rapid and robust increase in CCK-BR sensor fluorescence, indicating activity-dependent CCK release in the hippocampus. Based on these findings, we deduced that hippocampal CCK release plays a critical role in hippocampus-dependent behavioral tasks.

We acknowledge that hippocampal neurons receive CCK-positive projections from multiple brain regions, making it technically challenging to isolate and monitor the precise source of CCK release in the CA1 area during behavioral tasks in vivo. One potential strategy to address this limitation is selective overexpression of CCK in CA3 neurons (e.g., AAV-CCK delivery), followed by assessment of CCK-BR sensor responses during hippocampal-dependent behaviors. We have added this discussion to the revised manuscript to clarify the source and functional relevance of CCK release during behavioral tasks.

“Besides, using a GPCR-based CCK-BR sensor combined with fiber photometry, our previous work demonstrated rapid, activity-dependent CCK release in the hippocampus during object-exploratory behavior, supporting a functional role for hippocampal CCK signaling in cognitive tasks (Su et al., 2023). Given that hippocampal neurons receive CCK-positive projections from multiple brain regions, it remains technically challenging to precisely identify the cellular source of CCK release in CA1 during behavior. Future studies employing selective CCK overexpression in CA3 neurons, together with CCK-BR sensor recordings, may help further delineate the contribution of CA3-derived CCK to hippocampal-dependent behaviors.” (Line 313 - line 321 in the revision).

Citation:

(1) Wang, J. L., Sha, X. Y., Shao, Y., Zhang, Z. H., Huang, S. M., Lin, H., ... & Sun, J. P. (2025). Elucidating pathway-selective biased CCKBR agonism for Alzheimer’s disease treatment. Cell.

(2) Zhang, N., Sui, Y., Jendrichovsky, P., Feng, H., Shi, H., Zhang, X., ... & He, J. (2024). Cholecystokinin B receptor agonists alleviates anterograde amnesia in cholecystokinin-deficient and aged Alzheimer's disease mice. Alzheimer's research & therapy, 16(1), 109.

(3) Su, J., Huang, F., Tian, Y., Tian, R., Qianqian, G., Bello, S. T., ... & He, J. (2023). Entorhinohippocampal cholecystokinin modulates spatial learning by facilitating neuroplasticity of hippocampal CA3-CA1 synapses. Cell Reports, 42(12).

https://doi.org/10.7554/eLife.109001.1.sa1

**Reviewer #3 (Public review):**

Summary:

Fengwen Huang et al. used multiple neuroscience techniques (transgenetic mouse, immunochemistry, bulk calcium recording, neural sensor, hippocampal-dependent task, optogenetics, chemogenetics, and interfer RNA technique) to elucidate the role of the excitatory cholecystokinin-positive pyramidal neurons in the hippocampus in regulating the hippocampal functions, including navigation and neuroplasticity.

Strengths:

(1) The authors provided the distribution profiles of excitatory cholecystokinin in the dorsal hippocampus via the transgenetic mice (Ai14::CCK Cre mice), immunochemistry, and retrograde AAV.

(2) The authors used the neural sensor and light stimulation to monitor the CCK release from the CA3 area, indicating that CCK can be secreted by activation of the excitatory CCK neurons.

(3) The authors showed that the activity of the excitatory CCK neurons in CA3 is necessary for navigation learning.

(4) The authors demonstrated that inhibition of the excitatory CCK neurons and knockdown of the CCK gene expression in CA3 impaired the navigation learning and the neuroplasticity of CA3-CA1 projections.

Weaknesses:

(1) The causal relationship between navigation learning and CCK secretion?

Thank you for pointing out this important issue. Previous studies have shown that CCK can be rapidly secreted during exploratory behaviors, as detected by the CCK-BR sensor. In parallel, CCK-positive neurons have been demonstrated to play a critical role in the precise execution of hippocampus-dependent spatial learning. Together, these findings suggest that exploratory behavior induces CCK secretion, which in turn contributes to the accuracy of hippocampal-dependent learning and memory processes. Based on this evidence, we propose that CCK secretion serves as a functional link between behavioral exploration and spatial learning. We have added these explanations in the revised manuscript to better clarify the causal relationship between behavioral exploration and CCK secretion:

“Besides, using a GPCR-based CCK-BR sensor combined with fiber photometry, our previous work demonstrated rapid, activity-dependent CCK release in the hippocampus during object-exploratory behavior, supporting a functional role for hippocampal CCK signaling in cognitive tasks (Su et al., 2023). Given that hippocampal neurons receive CCK-positive projections from multiple brain regions, it remains technically challenging to precisely identify the cellular source of CCK release in CA1 during behavior. Future studies employing selective CCK overexpression in CA3 neurons, together with CCK-BR sensor recordings, may help further delineate the contribution of CA3-derived CCK to hippocampal-dependent behaviors.” (Line 313 - line 321 in the revision)

(2) The effect of overexpression of the CCK gene on hippocampal functions?

We thank the reviewer for this comment. In fact, an earlier study from our laboratory demonstrated that intraperitoneal injection of exogenous CCK-4 significantly improved performance in hippocampus-dependent spatial learning tasks in both CCK gene knockout (CCK-KO) mice and Alzheimer’s disease (AD) mouse models. These findings suggest that enhancing CCK signaling can ameliorate hippocampal dysfunction at both the behavioral and synaptic plasticity levels (Zhang et al., 2024; Wang et al., 2025). Accordingly, although direct genetic overexpression of CCK in the hippocampus has not yet been extensively characterized, the observed benefits of exogenous CCK delivery support the notion that increased CCK availability positively modulates hippocampal function and spatial learning. We have cited this study in the revised manuscript to support this interpretation.

“Interestingly, an earlier study demonstrated that intraperitoneal injection of exogenous CCK-4 significantly improved performance in hippocampus-dependent spatial learning tasks in both CCK gene knockout (CCK-KO) mice and Alzheimer’s disease (AD) mouse models (Zhang et al., 2024). These findings suggest that enhancing CCK signaling can ameliorate hippocampal dysfunction at both the behavioral and synaptic plasticity levels.” (Line 291 - line 297 in the revision)

(3) What are the functional differences between the excitatory and inhibitory CCK neurons in the hippocampus?

In the hippocampus, CCK-expressing neurons consist of two major populations with distinct functions: excitatory (glutamatergic) and inhibitory (GABAergic) neurons. Excitatory CCK neurons are relatively sparse and intermingled with pyramidal cells. By releasing glutamate, they directly contribute to excitatory transmission and are thought to participate in synaptic plasticity and information processing related to learning and memory. In contrast, inhibitory CCK neurons are more abundant and include well-characterized interneuron subtypes such as CCK-positive basket cells. These neurons release GABA and primarily target the perisomatic region of pyramidal neurons, providing strong control over neuronal firing. Notably, inhibitory CCK interneurons are highly sensitive to neuromodulatory signals, particularly endocannabinoids via CB1 receptors, enabling dynamic regulation of inhibitory tone and network activity. Together, excitatory CCK neurons mainly support hippocampal excitation and plasticity, whereas inhibitory CCK neurons regulate network dynamics and spike timing. As the focus of the present study is on excitatory CCK neurons, a detailed comparison between these two populations was not included in the original manuscript.

(4) Do CCK sources come from the local CA3 or entorhinal cortex (EC) during the high-frequency electrical stimulation?

Thank you for this insightful comment. Our data indicate that the CCK detected during high-frequency stimulation originates from CA3 neurons rather than the entorhinal cortex (EC). As shown in Figure 2, we used an optogenetic approach combined with a GPCR-based CCK sensor to selectively examine CCK release from the CA3-CA1 pathway. ChrimsonR was specifically expressed in CA3 neurons projecting to CA1, restricting light stimulation to CA3 axon terminals. In parallel, the CCK sensor was locally expressed in CA1, allowing real-time detection of CCK release at CA3 presynaptic sites. High-frequency light stimulation robustly evoked CCK signals in CA1, demonstrating activity-dependent CCK release from CA3 terminals. Importantly, EC inputs were neither genetically targeted nor optically stimulated in this experiment, excluding the EC as a source of the detected CCK. Together, these results support the conclusion that CCK released during high-frequency stimulation is derived from local CA3 projections to CA1. Similarly, as the focus of the present study is on excitatory CCK neurons in the CA3 area, a detailed comparison between these two CCK sources was not included in the original manuscript.

Citation:

(4) Wang, J. L., Sha, X. Y., Shao, Y., Zhang, Z. H., Huang, S. M., Lin, H., ... & Sun, J. P. (2025). Elucidating pathway-selective biased CCKBR agonism for Alzheimer’s disease treatment. Cell.

(5) Zhang, N., Sui, Y., Jendrichovsky, P., Feng, H., Shi, H., Zhang, X., ... & He, J. (2024). Cholecystokinin B receptor agonists alleviates anterograde amnesia in cholecystokinin-deficient and aged Alzheimer's disease mice. Alzheimer's research & therapy, 16(1), 109.

(6) Su, J., Huang, F., Tian, Y., Tian, R., Qianqian, G., Bello, S. T., ... & He, J. (2023). Entorhinohippocampal cholecystokinin modulates spatial learning by facilitating neuroplasticity of hippocampal CA3-CA1 synapses. Cell Reports, 42(12).
